# Are Invasive Group A Streptococcal Infections Preventable by Antibiotic Therapy?: A Collaborative Retrospective Study

**DOI:** 10.1097/INF.0000000000004403

**Published:** 2024-09-16

**Authors:** Rahel Erlacher, Nicole Toepfner, Svenja Dressen, Reinhard Berner, Annemarie Bösch, Tobias Tenenbaum, Ulrich Heininger

**Affiliations:** * From the Department of Paediatric Infectious Diseases and Vaccinology, University Children’s Hospital Basel (UKBB), Basel, Switzerland; † Department of Pediatrics, Faculty of Medicine and University Hospital Carl Gustav Carus, Technische Universität Dresden, Dresden, Germany; ‡ Child and Adolescent Medicine, Sana Children’s Hospital Lichtenberg, Academic Teaching Hospital Charité-Universitätsmedizin Berlin, Germany.

**Keywords:** group A streptococcus, invasive, children, antibiotics

## Abstract

**Background:**

In winter 2022/2023, a resurgence of invasive group A streptococcal (iGAS) infections in children was observed in Europe, including Germany and Switzerland. While a simultaneous increase in consultations for scarlet fever and pharyngitis was reported in England, leading to the recommendation to treat any suspected GAS disease with antibiotics, guidelines in Germany and Switzerland remained unchanged. We aimed to investigate whether this policy was appropriate.

**Methods:**

We conducted a retrospective multicenter study of children hospitalized for invasive GAS disease between September 2022 and March 2023 in pediatric departments in Dresden and Berlin (Germany) and Basel (Switzerland). We reviewed medical records and conducted structured telephone interviews to analyze whether suspected GAS infections with or without antibiotic treatment were reported prehospitalization.

**Results:**

In total, 63 patients met the inclusion criteria (median age 4.2 years, 57% males); however, clinical information was not complete for all analyzed characteristics; 32/54 (59%) had ≥1 physician visit ≤4 weeks prehospitalization. In 4/32 (13%) patients, GAS disease, that is, tonsillitis or scarlet fever, was suspected; 2/4 of them received antibiotics, and a positive rapid antigen test for GAS was documented in 1 of them.

**Conclusions:**

A small minority of patients had suspected GAS infection within 4 weeks before iGAS disease. These data suggest that there is little opportunity to prevent iGAS disease by antibiotic therapy, because in most patients—even if seen by a physician—there was either no evidence of GAS disease or when GAS disease was suspected and treated with antibiotics, consecutive invasive GAS disease was not prevented.

*Streptococcus pyogenes* [group A streptococcus, (GAS)] is a gram-positive bacterium that causes a wide variety of diseases. In children, mild to moderate infections such as tonsillopharyngitis, scarlet fever, or impetigo are common. However, in rare cases, GAS can be invasive and cause life-threatening conditions such as sepsis, meningitis, or necrotizing fasciitis, which require immediate antibacterial treatment and sometimes surgery.^1^

During the COVID-19 pandemic, a decline in several infectious diseases,^2^ including GAS infections,^3^ was observed. In the 2022/2023 winter season, WHO reported a resurgence of local and invasive GAS diseases in several European countries, with children under 10 years of age being the most affected age group.^4^ This increase raised general concern because of the severity of many cases and related fatalities.

Recently, before the pandemic, the previous paradigm of treating all patients with a sore throat and suspected GAS infections, such as tonsillopharyngitis, with an antibiotic had been abandoned in many countries including Germany^5^ and Switzerland.^6^ With this background, the resurgence of invasive GAS infections raised the question of whether every suspected GAS tonsillopharyngitis should be treated with an antibiotic to prevent invasive GAS disease in these patients. Professional societies, however, discouraged a change of recommendations.^7,8^

The aim of this study was to analyze whether there is retrospective evidence that invasive GAS disease could have been prevented if the respective patients had been presented to a physician before and if those with suspected GAS disease, such as tonsillopharyngitis had been treated with antibiotics. Therefore, we set out to investigate if patients with invasive GAS disease had presented to the health care system with GAS infections shortly before developing invasive disease and if these visits were missed opportunities for antibiotic treatment.

## METHODS

### Study Design

We conducted a retrospective multicenter study of patients who were treated for invasive GAS disease in 3 pediatric institutions: University Children’s Hospital Basel UKBB, Switzerland (study site A); the Departments of Pediatrics, University Hospital Carl Gustav Carus, Dresden, Germany (study site B); and Sana Children’s Hospital Lichtenberg, Berlin, Germany (study site C).

### Participants

Patients under 18 years of age hospitalized with invasive GAS disease in 1 of the 3 collaborating hospitals between September 01, 2022, and March 31, 2023 (Basel and Dresden) or May 31, 2023 (Berlin) were eligible for inclusion.

Invasive GAS disease was defined as the isolation of GAS from a normally sterile body site by bacterial culture and/or nucleic acid detection (polymerase chain reaction). Sterile sites included blood, cerebrospinal fluid, aspirate from pleura, joint, peritoneal or pericardial fluid, bone or muscle tissue or deep tissue from an internal body site (surgical sampling). If the treating physician’s diagnosis was necrotizing fasciitis or streptococcal toxic shock syndrome, isolation of GAS from a nonsterile site (eg, pharynx) was also considered as proof of invasive GAS disease.^9^

### Data Collection

Relevant data were extracted from electronic medical records at each hospital and managed using the REDCap electronic data capture system.^10^ In the absence of information on physician visits due to potential noninvasive GAS disease in the 4 weeks before hospital admission, we conducted structured telephone interviews with the families and, if necessary, with the patient’s pediatrician (Text, Supplemental Digital Content 1, http://links.lww.com/INF/F565).

### Statistical Analysis

Baseline data were summarized using descriptive statistics. Due to the limited number of patients, no analyses stratified by the study center were performed.

### Ethics

In this retrospective study, informed consent for phone interviews was obtained from the parents to complete nondocumented data. Approval was granted by the ethical committees in Dresden (BO-EK-252062023) and Berlin. At the study site in Basel, after consultation with the local ethics committee, approval was granted to include patients with existing general consent (EKNZ 2023-00696).

## RESULTS

### Patient Characteristics and Clinical Findings on Admission

**FIGURE 1.**
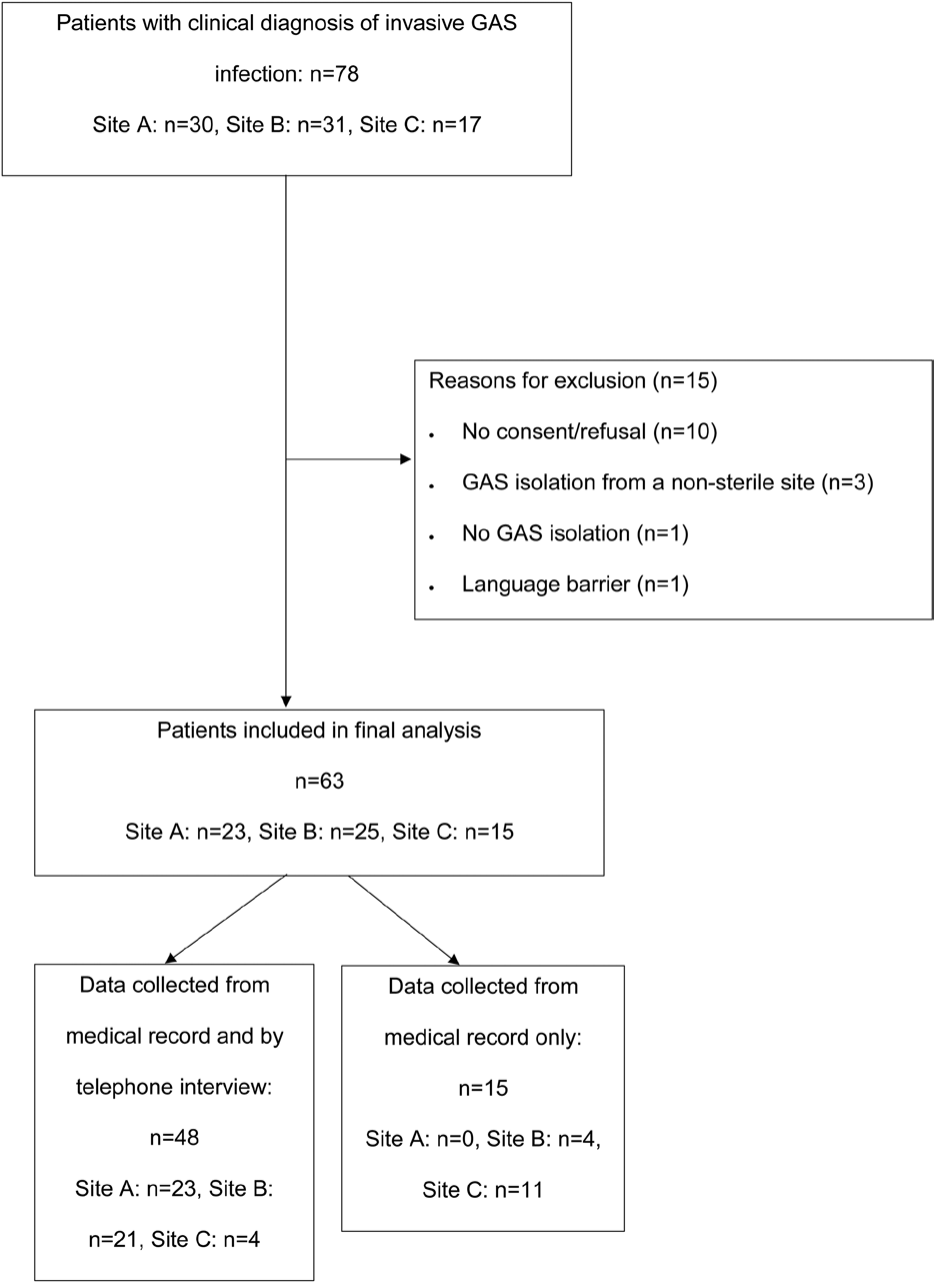
Flowchart of patient selection.

We identified a total of 78 patients hospitalized for invasive GAS, of which 63 fulfilled the inclusion criteria (Fig. 1).

Patient characteristics are presented in Table 1. Denominators vary because the available information was not complete for all patients. Six (10%) of 62 had an underlying disease but none was immunosuppressed. The most common clinical feature of invasive GAS disease was fever (95%), with a median duration of 4 days and a range of 0 to more than 10 days before admission. Signs and symptoms suggestive of local GAS infections, such as sore throat or typical rash for scarlet fever, were less common than unspecific signs and symptoms (Table 1).

**TABLE 1.** Characteristics and Clinical Presentation on Hospital Admission

Characteristics	n/n Known (%)
Median age: 4.2 years (IQR 1.8–6.9)	63/63 (100)
Age range: 3 months–14 years	63/63 (100)
Male sex	36/63 (57)
Chronic underlying disease	6/62 (10)
Immunosuppression	0/61 (0)
Fever duration: median 4 days (IQR 1–5)	59/63 (94)
Signs and symptoms*	
Fever (≥38 °C)	56/59 (95)
Cough and/or rhinitis	29/55 (53)
Vomiting and/or diarrhea	18/56 (32)
Limping and/or movement restrictions	12/52 (23)
Sore throat	11/48 (23)
Scarlet fever	7/56 (13)
Exanthema (unspecific)	6/57 (11)
Diagnosis of invasive GAS†	
Mastoiditis	16/63 (25)
Bloodstream infection (sepsis, bacteremia)	15/63 (24)
Pneumonia	14/63 (22)
Streptococcal Toxic Shock syndrome	12/63 (19)
Skin and soft tissue infection	11/63 (17)
Other clinical syndromes‡	10/63 (16)
Osteoarticular infection	9/63 (14)
Meningitis	3/63 (5)

* In most patients >1 symptom was present.

† 21 patients had >1 diagnosis of invasive GAS.

‡ Necrotizing fasciitis (n = 1), lymphadenitis colli (n = 3), para-/peritonsillar abscess (n = 3), pyomyositis (n = 3).

GAS, group A streptococcal infection.

Seven (16%) of 43 patients with iGAS disease had a known contact with GAS, usually in a family member, but none had contact with a case of iGAS disease.

### Clinical and Microbiologic Diagnosis

The predominant presentations of invasive GAS disease were bloodstream infections, pneumonia (mostly with pleura empyema; 12/14 cases) and mastoiditis (Table 1). About 21 (33%) of 63 patients had more than 1 invasive GAS disease manifestation.

Invasive GAS disease was confirmed by the isolation of GAS from the following specimens of sterile body sites: surgical soft tissue samples, that is, abscesses (n = 25); positive cultures of blood (n = 18); pleural fluid (n = 12); bone samples (n = 6); joint fluid (n = 5); muscle tissue (n = 4); and cerebrospinal fluid (n = 1). In 10 patients GAS was isolated from 2 body sites.

In 3 patients with the clinical presentation of streptococcal toxic shock syndrome, GAS was only isolated from a nonsterile site, that is, the pharynx (n = 3).

### Preadmission Physician Visits

Information on preadmission physician contact was available for 54 (86%) of 63 patients. Of these, 32 (59%) had a total of 42 physician visits (1 visit: n = 24; 2 visits: n = 6; 3 visits: n = 2) within 4 weeks before hospitalization (Table 2). However, only 4 (13%) of 32 patients with physician visits presented with a disease compatible with GAS infection: 3 with scarlet fever and 1 with tonsillopharyngitis (patients 1–4 in Table 2). Two of these 4 patients received a rapid antigen test for GAS, of which 1 was positive, and 2 of them were treated with an antibiotic.

**TABLE 2.** Details on 42 Pre-admission Physician Visits in 32 Patients with Invasive GAS Disease

Patient Number	Clinical Diagnosis	Day of Visit*	Rapid Antigen Test for GAS	Antibiotic Treatment*
1	Scarlet fever	−4 days	Unknown	Penicillin (−4 until 0)
2	1. Scarlet fever (unknown if with or without physician contact)	−14 days	Unknown	Unknown
	2. Gastroenteritis	−2 days	No	No
3	1. Upper respiratory tract infection	−14 days	Unknown	No
	2. Scarlet fever, tonsillitis	−12 days	Yes (positive)	Amoxicillin clavulanate (−12 until −6)
	3. Tonsillitis, otitis media	−6 days	No	Amoxicillin clavulanate (−6 until −5)
4	1. Pharyngitis	−5 days	No	No
	2. Tonsillitis	−3 days	Yes (negative)	No
5	Pharyngitis	−18 days	No	No
6	Upper respiratory tract infection	−27 days	No	No
7	Upper respiratory tract infection	−14 days	No	No
8	Upper respiratory tract infection	−12 days	No	No
9	Upper respiratory tract infection	−3 days	Unknown	No
10	Upper respiratory tract infection	−2 days	No	No
11	Upper respiratory tract infection	−2 days	No	No
12	Upper respiratory tract infection	−1 day	No	No
13	1. Upper respiratory tract infection	−12 days	No	No
	2. Upper respiratory tract infection	−5 days	No	No
14	1. Upper respiratory tract infection	−17 days	No	No
	2. Lymphadenitis colli	−16 days	No	Amoxicillin clavulanate (−16 until −11)
	3. Lymphadenitis colli	−10 days	Unknown	No
15	1. Other reason than infectious disease	−7 days	No	No
	2. Upper respiratory tract infection	−3 days	Yes (negative)	No
16	1. Other reason than infectious disease	−6 days	No	No
	2. Upper respiratory tract infection	−2 days	No	No
17	1. Upper respiratory tract infection, otitis media	−12 days	No	Amoxicillin clavulanate (−12 until −11)
	2. Otitis media	−11 days	No	Change to Cefclor (−11 until −4)
18	Otitis media	−28 days	No	Amoxicillin (−28 until −21)
19	Otitis media	−21 days	Unknown	Antibiotic, unknown product (−21 until −16)
20	Otitis media	−12 days	No	No
21	Otitis media	−3 days	No	No
22	Pneumonia	−14 days	No	Amoxicillin clavulanate (−14 until −6)
23	Bronchitis	−3 days	No	No
24	Bronchitis	−1 day	Yes (negative)	No
25	Gastroenteritis	−2 days	No	No
26	Varicella zoster virus infection	−3 days	No	No
27	Varicella zoster virus infection	−3 days	No	No
28	Ancle pain of unknown origin	−8 days	No	No
29	Unknown (symptomatic treatment)†	−5 days	Unknown	No
30	Unknown (symptomatic treatment)†	−2 days	Unknown	No
31	Unknown†	−14 days	No	No
32	Unknown†	−1 day	Unknown	Unknown

* In days before hospitalization for iGAS disease.

† Diagnosis not documented in patient records.

## DISCUSSION

There are 2 main findings in this study. First, the great majority (87%) of patients did not have any evidence for preceding local GAS disease within a 4-week window leading to a physician visit where prescription of an antibiotic treatment theoretically could have prevented their invasive GAS disease. Second, in 2 of the 4 patients with preceding suspected GAS infection, the disease was severe enough that an antibiotic was prescribed but this did not prevent consecutive invasive GAS disease. There was 1 patient with suspected GAS tonsillopharyngitis who was not treated with an antibiotic, but due to a negative rapid antigen test for GAS, this cannot truly be considered as a missed treatment opportunity.

The portal of entry for invasive GAS infections is often unclear, and invasive dissemination from the throat is still under debate.^11,12^ We found 3 other small pediatric studies where GAS-compatible diseases before invasive GAS infections were described. In a study from France,^13^ osteoarticular infections due to GAS were analyzed, and a preceding rhino-pharyngeal GAS infection was present in 7 (35%) of 20 cases. In a study from Australia,^14^ 15 (54%) of 28 children had been seen as out-patients within 48 hours before the onset of their invasive GAS disease, but only 1 (ie, 4% of the total) of them had local GAS disease (pyoderma) within the month before. In contrast, a study on invasive GAS infections in Greece^15^ reported that 19 (20%) of 96 pediatric patients had tonsillopharyngitis in the 4 weeks before hospitalization. Unfortunately, no data about antibiotic treatment of preceding GAS disease is provided in any of these studies. There are 2 other pediatric studies reporting symptoms suggestive of GAS disease, such as sore throat, before invasive GAS disease in 4%^16^ and 18%,^17^ respectively, but the time interval between GAS infection and invasive GAS diseases is not mentioned.

The only other data we are aware of that also investigated whether the invasive GAS disease may have been prevented by antibiotic therapy of preceding GAS-compatible illness is reported from the pediatric children’s hospital in Bern, Switzerland. The authors found that 5 (10%) of 51 children had a physician visit because of pharyngitis and/or scarlet fever within >24 hours before invasive GAS hospitalization. Like our study, only 2 of these 5 patients (ie, 4% of the total) did not receive an antibiotic^18^; one could argue that these 2 instances had been missed opportunities to prevent later hospitalization for invasive GAS.

Our study has some limitations, mainly its retrospective design and the limited number of patients. Another limitation is a possible recall bias regarding the information obtained from the parents through structured telephone interviews. To reduce this bias, we first interviewed the parents and if there was uncertainty, we called the treating pediatrician for the missing information. Despite the use of several available data sources (medical records, parents and pediatricians), the completeness of the data varied, which may limit interpretation of our findings. However, with regards to the main study objective, we believe that we did not miss a meaningful number of missed treatment opportunities for local GAS disease preceding invasive GAS disease. Strengths of the study are the multidisciplinary approach with 3 different pediatric centers and the focus on the association of GAS-compatible diseases preceding invasive GAS.

## CONCLUSIONS

The great majority of patients did not have a missed opportunity for antibiotic treatment preceding their iGAS disease. Therefore, based on our study and considering all limitations, withholding antibiotic treatment for suspected local GAS infections does not seem to be a risk factor for consecutive invasive GAS disease. The invasive GAS disease appears to be an independent event rather than following the preceding local GAS disease. Therefore, wisely chosen outpatient antibiotic treatment of suspected or proven noninvasive GAS disease in children remains justified and should be reinforced by current recommendations even in time periods of surging iGAS infections.

## Supplementary Material


