# Supplementary material for: Are Invasive Group A Streptococcal Infections Preventable by Antibiotic Therapy?: A Collaborative Retrospective Study
Source: Pediatr Infect Dis J. 2024 Sep 16;43(10):931–5. doi: 10.1097/INF.0000000000004403 (PMC11407772; doi:10.1097/INF.0000000000004403)
Supplement: Supplementary file 1 [file inf-43-0931-s001.docx]

**Telephone interview with parents**

Hello Ms/Mr xxxxx (last name). My name is Rahel Erlacher, I am a doctor from the University of Basel Children's Hospital (UKBB). Do you please have 5 minutes? (When else may I call again?)

-------------------------------------------------- -------------------------------------------------- ---------------------------------

Version A (phone call regarding general patient consent):

We have already had contact based on your consent for research and you have also received a cover letter from us stating that we will contact you once by telephone if routine data is possibly incomplete.

Version B (if there is already a general consent without prior telephone contact)

I'm contacting you as a member of Prof. Heininger's infectious disease team from the UKBB. You were with us in the children's hospital in xxx (autumn/winter/spring) with xxxxx (child's first name) because of xxxx (illness). The bacterium that caused the illness in your son/daughter is called group A streptococcus. We have been seeing an increasing number of such cases since last fall and are now evaluating them, and we are particularly interested in the medical history before the hospital stay. I would like to ask you xxx (missing information):

-------------------------------------------------- -------------------------------------------------- ---------------------------------

From an infectious disease perspective, I still have 2 short questions about the history before the hospital stay:

**B) Do you remember whether xxx (child's first name) was sick in the 4 weeks before you came to the hospital?**

*Ask for specific details on:*

- fever
- runny nose/cough
- pain
- vomiting/diarrhea
- sore throat
- limited mobility
- skin rash
- skin swelling and redness
- other symptoms?

**C) Did you see a doctor with your child in the last 4 weeks before admission to hospital?** **If yes, ask for specific details on:**

- Angina
- middle ear infection
- Scarlet fever
- Impetigo contagiosa
- UTI
- Cellulitis
- pneumonia
- Other diagnosis?
- Was a throat Strep A swab taken? Result?
- Was antibiotic treatment given? If yes, which product and for how long?

Thank you very much for your collaboration and taking the extra time. We wish you and your child all the best and, above all, good health for the future.

**Telephone interview with patient’s private pediatrician**

Hello Ms/Mr xxxxx (pediatrician). My name is Rahel Erlacher, I'm a physician calling from the University of Basel Children's Hospital (UKBB). Do you please have 2-3 minutes? (When else can I call again?)

I am contacting you regarding the following patient xxxx (name, date of birth ).

I am a member of Prof. Heininger's infectious disease team and we are interested in the medical history of children with invasive streptococcal A infections as part of a retrospective study.

xxxx family (patient's last name) told me that they had seen you in the 4 weeks before the hospitalization. Can you please give me some specific details about this visit in your office

*(check items from following list)*.

- angina
- middle ear infection
- scarlet fever
- impetigo contagiosa
- UTI
- cellulitis
- pneumonia
- other diagnosis?
- Was a throat Strep A swab taken? Result?
- Was antibiotic treatment given? If yes, which product and for how long?

Thank you very much for your cooperation and taking the extra time.
